# Caring for carers: A virtual psychosocial supervision intervention to improve the quality and sustainability of mental health and psychosocial support in humanitarian contexts

**DOI:** 10.1192/j.eurpsy.2022.2295

**Published:** 2022-09-01

**Authors:** R. Wells, C. Acartuk, F. Almeamari, M. Alokoud, A. Beetar, H. Eldardery, M. Elshazly, O. Faruk, M.R. Ginem, D. Hadzi-Pavlovic, Z. Ilkkurşun, S. Jahan, R. Joshi, L. Klein, L. Kurdi, G. Kurt, C. Mastrogiovanni, M. Mozumder, S. Lekkeh, S. Némorin, K. Nicholson Perry, M. Orabi, J. Qasim, Z. Steel, M. Tavakol, H. Ullah, E. Uygun, S. Wong, L. (Fischer) Yan, R. Said Yousself, A. Zarate, S. Rosenbaum

**Affiliations:** 1 UNSW Sydney, Faculty Of Medicine, Sydney, Australia; 2 Koç University, Department Of Psychology, Istanbul, Turkey; 3 Hope Revival Organization, Hope Revival Organization, Gaziantep, Turkey; 4 Istanbul Şehir University, Department Of Psychology, Istanbul, Turkey; 5 Independent consultant, N/a, Cairo, Egypt; 6 University of Dhaka, Department Of Clinical Psychology, Dhaka, Bangladesh; 7 NSW Service For The Treatment And Rehabilitation Of Torture And Trauma Survivors (STARTTS), Nsw Service For The Treatment And Rehabilitation Of Torture And Trauma Survivors (startts), Carramar, Australia; 8 Australian College of Applied Psychology (ACAP), Discipline Of Psychological Science, Sydney, Australia; 9 Hope Revival Organization, Hope Revival Organization, Northwest Syria, Syria; 10 Isık University, Department Of Psychology, Istanbul, Turkey; 11 Independent consultant, N/a, Cox’s Bazar, Bangladesh; 12 Bilgi University, Trauma And Disaster Mental Health, Istanbul, Turkey; 13 John Hopkins University, Department Of Mental Health, Baltimore, United States of America; 14 University of Denver, Graduate School Of Social Work & Josef Korbel School For International Studies, Denver, United States of America

**Keywords:** humanitarian, MHPSS, supervision, online

## Abstract

**Introduction:**

Mental health and psychosocial support (MHPSS) staff in humanitarian settings have limited access to clinical supervision and are at high risk of experiencing burnout. We previously piloted an online, peer-supervision program for MHPSS professionals working with displaced Rohingya (Bangladesh) and Syrian (Turkey and Northwest Syria) communities. Pilot evaluations demonstrated that online, peer-supervision is feasible, low-cost, and acceptable to MHPSS practitioners in humanitarian settings.

**Objectives:**

This project will determine the impact of online supervision on i) the wellbeing and burnout levels of local MHPSS practitioners, and ii) practitioner technical skills to improve beneficiary perceived service satisfaction, acceptability, and appropriateness.

**Methods:**

MHPSS practitioners in two contexts (Bangladesh and Turkey/Northwest Syria) will participate in 90-minute group-based online supervision, fortnightly for six months. Sessions will be run on zoom and will be co-facilitated by MHPSS practitioners and in-country research assistants. A quasi-experimental multiple-baseline design will enable a quantitative comparison of practitioner and beneficiary outcomes between control periods (12-months) and the intervention. Outcomes to be assessed include the Kessler-6, Harvard Trauma Questionnaire and Copenhagen Burnout Inventory and Client Satisfaction Questionnaire-8.

**Results:**

A total of 80 MHPSS practitioners will complete 24 monthly online assessments from May 2022. Concurrently, 1920 people receiving MHPSS services will be randomly selected for post-session interviews (24 per practitioner).

**Conclusions:**

This study will determine the impact of an online, peer-supervision program for MHPSS practitioners in humanitarian settings. Results from the baseline assessments, pilot evaluation, and theory of change model will be presented.

**Disclosure:**

No significant relationships.

